# 1LocusSim a mobile-friendly simulator for teaching population genetics

**DOI:** 10.1093/bioadv/vbad087

**Published:** 2023-07-05

**Authors:** Antonio Carvajal-Rodríguez

**Affiliations:** Department of Biochemistry, Genetics and Immunology, Facultad de Biologia, Centro de Investigación Mariña (CIM), University of Vigo, Vigo 36310, Spain

## Abstract

**Motivation:**

Biology students often struggle with the fundamental concepts of evolutionary genetics, including genetic drift, mutation and selection. To address this problem, 1LocusSim was developed to simulate the interaction of different factors, such as population size, mutation, selection and dominance, to study their effect on allelic frequency during evolution. With 1LocusSim, students can compare theoretical results with simulation outputs and solve and analyze different problems of population genetics. The 1LocusSim web has a responsive design which means that it has been specifically designed to be used on smartphones.

**Results:**

To demonstrate its use, I review the classical overdominance model of population genetics and highlight a characteristic that is often not explicitly stated. Specifically, it is emphasized that the equilibrium of the model does not depend on the homozygous selection coefficients but rather on the ratio of the selection coefficients. This is already clear from the classical formula but maybe not so much for students. Also it implies that the equilibrium can be expressed solely in terms of the dominance coefficient *h*. To verify this theoretical prediction, I utilize the simulator and calculate the equilibrium for the well-known case of sickle cell anemia. By utilizing this tool, students can learn at their own pace and convenience, anywhere and anytime.

**Availability and implementation:**

1LocusSim if freely available at https://1LocusSim-biosdev.pythonanywhere.com/. Website implemented under the Bottle micro web-framework for Python, with all major browsers supported.

**Supplementary information:**

Supplementary data are available at *Bioinformatics Advances* online.

## 1 Introduction

Smartphones offer new learning opportunities and provide approaches to foster the development of critical skills for learners in the 21st century. Smartphones have become an ingrained aspect of students’ lives, making it a comfortable medium for learning. One of the key benefits of the smartphone is its portability, allowing students to access learning content anytime, anywhere. With learning available at their fingertips, students can expand their knowledge without interruption. The prevalence of smartphones in households of all demographics makes it a readily available and accessible tool for learning. Here, I present a one-locus population genetics simulator called 1LocusSim. Unlike other teaching software, 1LocusSim is a wep app so it is not limited to a specific platform or operating system, as it is available in the cloud and does not require installation. It can be accessed from any device, including smartphones, for which it has been specially designed, and comes with a set of companion web pages that include a glossary of basic concepts, formulas, examples and exercises to enhance the students’ learning experience. Therefore, students only need to type the web address (1LocusSim-biosdev.pythonanywhere.com) from their preferred device to run the program.

As an example of the use of the simulator, we will review the basics of the overdominance model to calculate the equilibrium value of the sickle cell gene and use 1LocusSim to perform simulations that confirm the theoretical result.

## 2 Two allele overdominance model

In an overdominance fitness model, the heterozygote has higher fitness than the corresponding homozygotes. Typically, this is represented as the heterozygote having fitness 1, while the A_1_A_1_ and A_2_A_2_ homozygotes have lower fitness given by 1-*s*_1_ and 1-*s*_2_, respectively. In a large population, the equilibrium value for that model does not require mutation (Crow and Kimura, 1970) and it depends only on the ratio *k* = *s*_2_/*s*_1_ of the selection coefficients (see Supplementary Equation S1 and the *overdominance* section of the Manual). Sometimes it is useful to express the overdominance model using the selection coefficient *s* and the dominance coefficient *h* from the classical selection model, as shown in Table 1 (and in online Manual Tables 2 and 3).

**Table 1.** Overdominance expressed from the model with selection coefficient *s* and dominance coefficient *h* (*s *>* *0, *h *<* *0 or *s *<* *0, *h *>* *1)

A_1_A_1_	A_1_A_2_	A_2_A_2_
1	1-*sh*	1-*s*
1/(1-*sh*)	1	(1-*s*)/(1-*sh*)

In this case, *h *=* *1/(1−*k*) and the equilibrium value (Supplementary Equation S2) can be expressed just in terms of the dominance coefficient (Caballero, 2020). For finite population sizes, Robertson (1962) showed that the effect of genetic drift is controlled by *N*(*s*_1_+*s*_2_)*f* where *f* is the equilibrium frequency of the lowest fit allele. Under our notation the condition in Robertson can be expressed as a function of |*Nsh*|. The higher this value, the lower the effect of genetic drift and vice versa.

## 3 Simulations

Since 1LocusSim is designed to be used both in classrooms and on mobile devices such as smartphones and tablets, it is important that simulation times do not exceed a couple of minutes even in the worst-case scenario. Therefore, the parameter values have been limited to ensure that the simulation runs within this time frame (please refer to the online *manual* for a more detailed explanation of parameter limits). Despite these limitations, we can use 1LocusSim to test the dependence of overdominance equilibrium solely on the *h* parameter and study as an example the sickle cell anemia (SCA) overdominance model.

Therefore, we first test whether the simulations behave as predicted for the overdominance model in general, such that the equilibrium values only depend on *h*. The simulation results shown in Supplementary Table S1, demonstrate that the equilibrium values are in good agreement with expectations and clearly depend on the *h* value as predicted (see also online Manual’s Table 4 and Figure 6). However, as expected, the lower the |*Nsh*| value the higher the noise due to genetic drift.

## 4 Overdominance equilibrium for a lethal allele: the SCA

SCA is a classical example of overdominance. In high-malaria regions of Africa, homozygotes for the non-SCA allele have a relative fitness of 0.85 (*s*_1_ = 0.15). The SCA allele can be considered a recessive lethal (*s*_2_ = 1) in regions without modern medical care (Hartl, 2020). Heterozygous individuals have fewer physiological effects of the SCA condition and partial resistance to malaria. Therefore, the overdominance model for the SCA implies, using our previous notation, *k* = *s*_2_/*s*_1_ = 6.7 and *h *=* *1/(1−*k*) = −0.175 and the expected equilibrium frequency of the SCA allele in the absence of mutation is *q*_eq_ = *h*/(2*h*−1) = 0.13.

Now, we can run simulations to calculate the equilibrium value of the SCA gene. Thus, we set the following parameters in LocusSim: *n *=* *1, *N = *10^4^, *q*_0_* *=* *0.01, *µ *=* *0, *h *=* *−0.175, *T *=* *10^4^ and run simulations under different selection coefficients (*s *=* *0.01, 0.1, 0.5 and 1). We repeat each simulation 10 times and compute the mean and standard deviation of the observed frequency *q*_obs_. Simulations confirmed that an equilibrium value close to 0.13 is obtained for *h *=* *−0.175 under different selection coefficients when |*Nsh*| is high enough (Table 2).

**Table 2.** Equilibrium values for the sickle cell gene after simulation

*s*	*h*	*|Nsh|*	*q*_eq_	*q*_obs_
0.01	−0.175	17.5	0.130	0.046 ± 0.049
0.1	−0.175	175	0.130	0.126 ± 0.014
0.5	−0.175	875	0.130	0.131 ± 0.000
1	−0.175	1750	0.130	0.129 ± 0.000

*Note*: Population size *N* = 10^4^, initial frequency *q*_0_ = 0.01, generations *T *=* *10^4^. Results are average of 10 runs.

*q*_eq_, expected equilibrium frequency; *q*_obs_, mean observed frequency.

In addition, the equilibrium with mutation (Bürger, 1998) was also obtained (Supplementary Equation S3) and the equilibrium predicted values confirmed by simulation (Supplementary Table S2).

## 5 Discussion

The 1LocusSim simulator usefulness has been demonstrated by reviewing the fundamentals of the overdominance model. Additional examples for mutation, drift and selection equilibriums jointly with exercises, and detailed explanations can be found in the online manual.

Many Biology students often feel a strong rejection toward mathematics and related knowledge. This is often attributed to difficulties in understanding abstract concepts, whether they are mathematical or from other fields, as well as a lack of awareness regarding its relevance in Biology. Moreover, the approach to knowledge acquisition in the field of Biology tends to have limited emphasis on quantitative aspects (Chiel *et al.*, 2010; Feser *et al.*, 2013; Rubinstein and Chor, 2014). Simulating basic population genetic models on smartphones can be a powerful learning aid that fosters critical skills and opens up new opportunities for Biology students. 1LocusSim enables students to learn at their preferred pace and convenience, anywhere and anytime.

## Supplementary Material

vbad087_Supplementary_Data

## Data Availability

No new data were generated or analysed in support of this research. And specifically the referred online manual tables and Figure are available at: https://acraaj.webs.uvigo.es/1LocusSim/1LocusSim_Seleccion_EN.html.
