# Supplementary material for: 1LocusSim a mobile-friendly simulator for teaching population genetics
Source: Bioinform Adv. 2023 Jul 5;3(1):vbad087. doi: 10.1093/bioadv/vbad087 (PMC10343943; doi:10.1093/bioadv/vbad087)
Supplement: vbad087_Supplementary_Data [file vbad087_supplementary_data.pdf]

Supplementary Appendix: 1LocusSim a mobile-friendly simulator for teaching population genetics

Two allele overdominance model

In an overdominance fitness model, the heterozygote has higher fitness than the corresponding homozygotes. Typically, this is represented as the heterozygote having fitness 1, while the A_1A_1 and A_2A_2 homozygotes have lower fitness given by 1-s_1 and 1-s_2 , respectively.

In a large population, the equilibrium value for that model does not require mutation and is (Crow and Kimura, 1970) $q_{eq} = s_1 / (s_1 + s_2)$. This equation can be rearranged as

$$q_{eq} = \frac{1}{\frac{(s_1+s_2)}{s_1}} = \frac{1}{(1+k)} \qquad \text{where } k = s_2/s_1 \text{ and } s_1>0,\ s_2>0 \qquad \text{(S1)}$$

Note that k is only defined in an overdominance scenario, i.e. only if s_1 and $s_2 > 0$. From (S1) is clear that the equilibrium depends on the ratio of the selection coefficients rather than on each coefficient. Different pairs s_1, s_2 may have the same equilibrium frequency provided the ratio remains constant. The equilibrium have different ranges depending on if k>1 ie. $w_{A1A1} > w_{A2A2}$, q_{eq} is in (0, 0.5) on the contrary, if k<1 ie. $w_{A1A1} < w_{A2A2}$, q_{eq} is in (0.5, 1). If k = 1, q_{eq} = 0.5.

The overdominance model can be expressed using the selection coefficient s and the dominance coefficient h from the classical selection model, as has been shown in Table 1 in the manuscript. The Table 1 presents an overdominance model when s>0, h<0 or s<0, h>1. Consequently we can express $k = s_2/s_1 = (h-1)/h$ or $h = 1/(1-k)$ and so the equilibrium value

$$q_{eq} = \frac{1}{(1+k)} = \frac{h}{2h-1} \qquad \text{(S2)}$$

It's worth noting that the equilibrium value is determined by the dominance coefficient rather than the selection coefficient (Caballero, 2020). This is because the equilibrium depends on the ratio of the homozygous coefficients, rather than the coefficients themselves. For finite population sizes (Robertson, 1962) showed that the effect of genetic drift is controlled by $N(s_1+s_2)f$ where f is the equilibrium frequency of the lowest fit allele. Under our notation the condition in Robertson can be

expressed as a function of |*Nsh*|. The higher this value, the lower the effect of genetic drift and *vice versa*.

Table S1. Overdominance: comparison of expected and observed equilibrium values.

**Model**	***s***	***h***	**|*Nsh*|**	q_{eq}	q_{obs}
$w_{A1A1} > w_{A2A2}$	0.01	-0.1	10	0.083	0.021±0.198
$w_{A1A1} > w_{A2A2}$	0.1	-0.1	10^2	0.083	0.074±0.028
$w_{A1A1} > w_{A2A2}$	1	-0.1	10^3	0.083	0.086±0.000
$w_{A1A1} > w_{A2A2}$	0.01	-0.5	50	0.25	0.272±0.036
$w_{A1A1} > w_{A2A2}$	0.1	-0.5	500	0.25	0.243±0.010
$w_{A1A1} > w_{A2A2}$	1	-0.5	5×10^3	0.25	0.251±0.000
$w_{A1A1} > w_{A2A2}$	0.01	-100	10^4	0.4975	0.498±0.000
$w_{A1A1} > w_{A2A2}$	0.1	-100	10^5	0.4975	0.498±0.000
$w_{A1A1} > w_{A2A2}$	1	-100	10^6	0.4975	0.496±0.000
$w_{A1A1} < w_{A2A2}$	-0.01	1.05	10^2	0.955	0.968±0.056
$w_{A1A1} < w_{A2A2}$	-1	1.05	10^4	0.955	0.954±0.01
$w_{A1A1} < w_{A2A2}$	-0.01	1.25	1.25×10^2	0.83	0.84±0.049
$w_{A1A1} < w_{A2A2}$	-1	1.25	1.25×10^4	0.83	0.836±0.01
$w_{A1A1} < w_{A2A2}$	-0.01	100	10^4	0.5025	0.506±0.000
$w_{A1A1} < w_{A2A2}$	-1	100	10^6	0.5025	0.503±0.000

Population size $N=10^4$, initial frequency $q_0 = 0.01$, number of generations $T = 10^4$. Results are average of 10 runs.

Equilibrium with overdominance and mutation

We can use the equation (2.5) in (Bürger, 1998) with $h<0$ to obtain the equilibrium value with overdominance for a lethal allele ($s=1$), mutation $\mu>0$ and negligible retromutation $\nu=0$. After some algebra manipulation we obtain

$$q_{eq}=\frac{|h|}{2(2|h|+1)}+\frac{\sqrt{h^2+4(2|h|+1)(|h|+1)\mu}}{2(2|h|+1)} \quad \text{(S3)}$$

Then if we substitute h=-0.175 the equilibrium value is

$$q_{eq}=\frac{0.175}{2.7}+\frac{\sqrt{0.0306+6.345\mu}}{2.7}$$

if $\mu=5\times10^{-8}$ (Melamed *et al.*, 2022) then

$$q_{eq}=\frac{0.175}{2.7}+\frac{\sqrt{0.0306+6.345\mu}}{2.7}\simeq 0.13$$

if μ=0.01 then q_{eq} = 0.178 and if μ=0.1 then q_{eq} = 0.367.

Table S2. Sickle cell anaemia equilibrium with mutation.

s	***h***	***μ***	q_{eq}	q_{obs}
1	-0.175	5×10^{-8}	0.130	0.130±0.000
1	-0.175	0.01	0.178	0.173±0.000
1	-0.175	0.1	0.367	0.347±0.000

Population size $N=10^4$, initial frequency q_0=0.01, q_{eq}: expected equilibrium frequency, q_{obs}: observed frequency after 10^4 generations. Results are average of 10 runs.

References

Bürger,R. (1998) Mathematical properties of mutation-selection models. *Genetica*, **102**, 279–298.

Caballero,A. (2020) Quantitative Genetics Cambridge University Press.

Crow,J.F. and Kimura,M. (1970) An Introduction to Population Genetics Theory Harper & Row, New York.

Melamed,D. *et al.* (2022) De novo mutation rates at the single-mutation resolution in a human HBB gene region associated with adaptation and genetic disease. *Genome Res*, **32**, 488–498.

Robertson,A. (1962) Selection for heterozygotes in small populations. *Genetics*.
